# Unusual Presentation of Eumycetoma (Madura Foot): A Case Report

**DOI:** 10.7759/cureus.104719

**Published:** 2026-03-05

**Authors:** Syed Muhammad Hayyan Nishat, Dima Ibrahim, Seema Oommen, Prathap c Potula

**Affiliations:** 1 Internal Medicine, Burjeel Medical City, Abu Dhabi, ARE; 2 Medicine and Health Sciences, Khalifa University, Abu Dhabi, ARE; 3 Transplant Infectious Diseases, Burjeel Medical City, Abu Dhabi, ARE; 4 Laboratory Science, Burjeel Medical City, Abu Dhabi, ARE; 5 Vascular Surgery, Burjeel Medical City, Abu Dhabi, ARE

**Keywords:** atypical mycetoma presentation, eumycetoma, extra-pedal mycetoma, madura foot, madurella mycetomatis, subcutaneous fungal infection

## Abstract

Mycetoma, commonly referred to as Madura foot, is a chronic granulomatous infection of the skin and subcutaneous tissues caused by fungi (eumycetoma) or filamentous bacteria (actinomycetoma). It typically presents in the foot following traumatic inoculation in endemic regions. Extra-pedal presentations are uncommon and may pose diagnostic and therapeutic challenges. We present the case of a 21-year-old previously healthy male patient with an unusual eumycetoma involving the posterior chest wall (back), an atypical anatomical site. The diagnosis was initially suspected clinically due to the discharge of black grains from the lesion and was subsequently confirmed by cytology, fungal culture, and molecular sequencing, which identified Madurella mycetomatis. This case highlights the importance of considering mycetoma in chronic, indolent subcutaneous lesions, particularly in endemic areas, even when lesions arise outside the commonly affected sites.

## Introduction

Mycetoma is a chronic, progressive, and deforming infection that thrives in arid and tropical climates, particularly in regions of Africa, the Middle East, and South Asia [1]. It is characterized by tumefaction, draining sinuses, and extrusion of grains composed of colonies of the causative organism [2]. Eumycetoma, most frequently caused by Madurella mycetomatis, accounts for a substantial proportion of cases in endemic zones [3]. Infection typically follows traumatic implantation of fungal elements from contaminated soil into exposed body parts, predominantly the foot, which accounts for over 70% of all cases [1].

Extra-pedal mycetoma is considered highly unusual. When it does occur, the trunk, back, or upper limbs may be involved, but such cases are often misdiagnosed because clinicians generally do not associate these locations with mycetoma [4]. The absence of the classic triad early in the disease course further contributes to delayed diagnosis and inappropriate management [4]. Early identification is essential because long-standing mycetoma can lead to extensive tissue destruction, disability, and significant morbidity [1].

## Case presentation

A 21-year-old previously healthy male patient presented with a four-year history of a gradually progressive swelling on the right upper back. He had previously resided in Sudan and reported occupational exposure through close contact with camels. The lesion was associated with intermittent pain and a sense of fullness but no history of fever, constitutional symptoms, or trauma. He denied any previous similar lesions, surgeries, or systemic illnesses. The swelling had slowly enlarged over the years, and the patient reported episodes of dark-colored discharge containing black granules within the preceding year. On examination, a multiloculated, firm, non-tender mass measuring approximately 4-5 cm in greatest dimension was noted over the right posterior and lateral chest wall. Several small sinus openings were visible, and a few extruded black grains were noted on pressure. There was no regional lymphadenopathy. Vital signs were stable, and the patient was afebrile. The patient appeared well and systemically asymptomatic.

Investigations

MRI of the chest revealed multiple, well-defined cystic lesions with internal septations within the subcutaneous tissue of the right posterior and lateral chest wall. The lesions demonstrated mixed T1 and heterogeneously hyperintense T2 signals, with mild peripheral enhancement upon contrast administration. The chest wall muscles, ribs, and thoracic cavity structures appeared preserved with no evidence of deep infiltration. The classic “dot-in-circle” sign, considered characteristic of mycetoma on MRI, was not clearly demonstrated in the available imaging. These features initially suggested possible lymphatic or veno-lymphatic malformation, given the presence of multi-septated cystic spaces.

**Figure 1:**
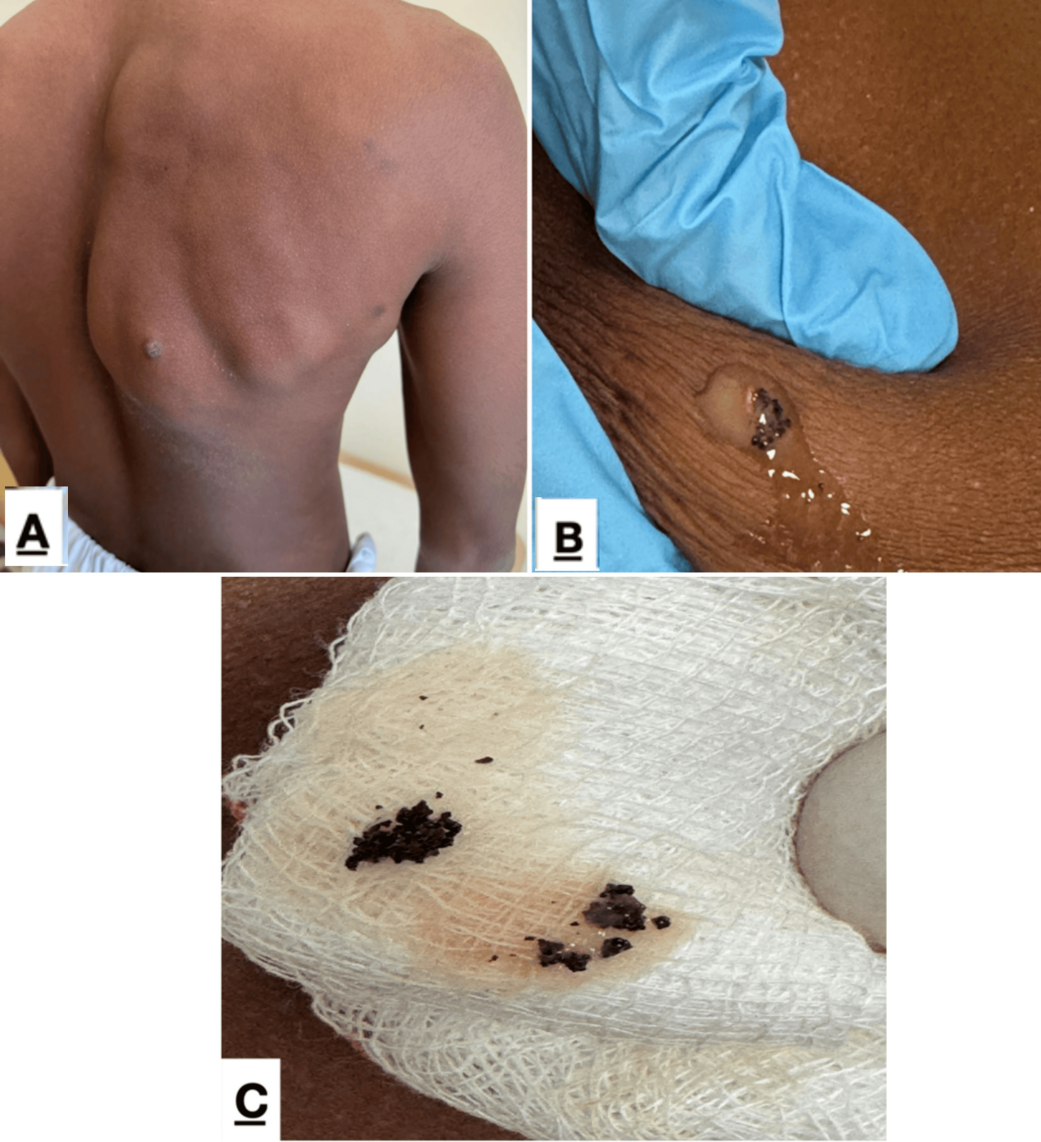
Clinical presentation and characteristic grains of eumycetoma (A) Posterior trunk showing localized subcutaneous swelling on the right upper back; (B) Close-up of lesion demonstrating purulent discharge and visible black grains; (C) Extruded black granules collected on gauze following expression from the lesion.

Aspiration performed in the outpatient setting yielded thick, viscous, dark-brown fluid containing black granules (Figure 1).

**Figure 2:**
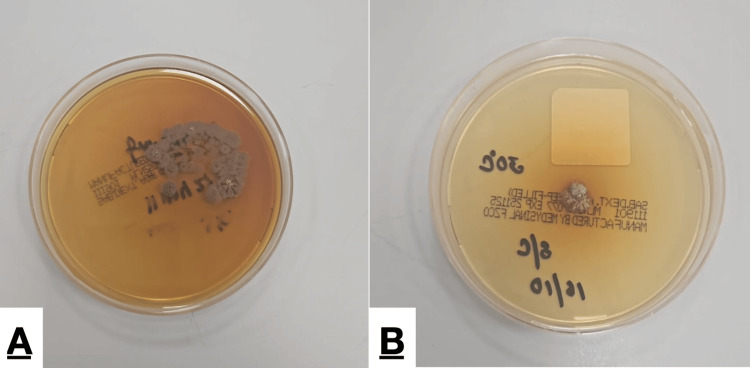
Colony growth on Sabouraud dextrose agar (A) Colony growth after 10 days of incubation showing dark folded colonies; (B) Colony growth demonstrating brown diffusible pigment.

This finding immediately raised suspicion for mycetoma. The granules were submitted for microbiological workup. They were cultured on Sabouraud’s dextrose agar and incubated at 37°C and at 30°C. Colonies grew slowly and were yellow to yellowish-brown with a leathery texture by the first week and later became brownish with a diffusible pigment with the texture being folded with age by around 10 days (Figure 2). 

The reverse of the colony was dark brown.

**Figure 3:**
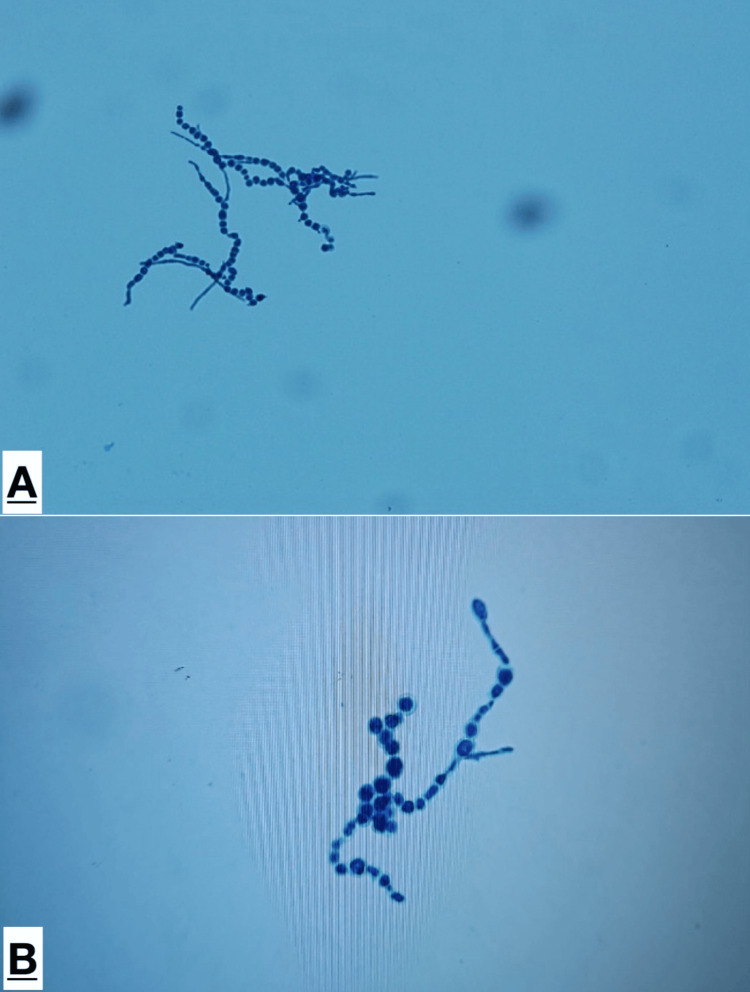
Lactophenol cotton blue preparation from growth on Sabouraud’s dextrose agar (A) Dematiaceous filamentous hyphae without conidiation; (B) Numerous chlamydoconidia-like, enlarged cells.

Microscopically, on lactophenol cotton blue preparations branching dematiaceous septate filamentous hyphae (1-6µm) with numerous chlamydoconidia-like, enlarged cells were seen. Hyphae appeared sterile with barely any conidiation as seen (Figure 3).

Growth at 30°C was absent. Cornmeal agar was not available in the laboratory and hence wasn’t used for culture and demonstrating phialides.

Considering the presence of black granules, growth at 37°C and presence of diffusible pigment being present, morphological diagnosis of Madurella mycetomatis was made thus differentiating it from Madurella grisea. The fungal isolate was subsequently sent for molecular identification. Internal transcribed spacer (ITS) and D1/D2 region sequencing re-confirmed the identification as Madurella mycetomatis. Sequence comparison analysis demonstrated 99.79% (ITS) and 99.83% (28S ribosomal RNA gene) similarity with reference sequences of Madurella mycetomatis type material (CBS 109801), confirming species-level identification. Hematological investigations revealed peripheral eosinophilia (absolute eosinophil count: 1000 cells/µL; reference value: 0-500 cells/µL). Although eosinophilia is not a specific feature of eumycetoma, mild elevation may be observed in chronic fungal infections due to ongoing inflammatory and immune responses.

Differential diagnosis

The initial clinical and radiological assessment generated a differential diagnosis that included lymphatic malformation, veno-lymphatic malformation, chronic cystic infection, and eumycetoma. The presence of black granules on aspiration, combined with cytological and microbiological confirmation, ultimately established the diagnosis of eumycetoma.

Treatment

The patient was commenced on oral itraconazole at a dose of 200 mg twice daily, which is the standard first-line antifungal therapy for eumycetoma caused by Madurella mycetomatis. Before diagnosis, surgical intervention, including sclerotherapy, had been considered under the assumption of a vascular malformation. However, once fungal grains were identified, sclerotherapy was deferred.

The case was discussed jointly by vascular surgery and infectious disease specialists. Given the lack of muscular, osseous, or thoracic involvement, a conservative medical approach was favored. The patient was counseled regarding the prolonged duration of antifungal therapy, with a planned minimum treatment duration of six to 12 months depending on clinical and radiological response, the need for adherence, and regular monitoring of liver function due to itraconazole’s potential hepatotoxicity.

Outcome and follow-up

Given the high clinical suspicion for mycetoma, empiric oral itraconazole therapy was commenced before microbiological confirmation. The patient was subsequently lost to follow-up and failed to attend scheduled outpatient reviews. Consequently, assessment of treatment response and long-term outcomes was not possible, which represents a limitation of this case report.

## Discussion

Eumycetoma remains a neglected tropical disease, often affecting young adults engaged in agricultural activity in endemic regions [5]. To our knowledge, very few published cases describe eumycetoma localized to the posterior chest wall, making this presentation particularly unusual. Although the foot is the most commonly affected site, extra-pedal presentations, such as the back, are exceedingly rare and often lead to misdiagnosis [4].

The patient reported carrying his backpack directly on his bare back during prolonged walks in Sudan. This activity may have led to the infection of the back through direct traumatic inoculation of pathogenic fungi from contaminated material on the backpack into abraded or broken skin.

The absence of typical clinical signs early in the disease confounds identification, and imaging alone is frequently insufficient to distinguish mycetoma from benign cystic lesions or vascular malformations [4]. MRI findings in mycetoma can include multiple small hyperintense cavities, the “dot-in-circle” sign, and septated lesions, though these may be subtle or absent in early or atypical cases [1]. In this patient, features mimicked lymphatic malformation, illustrating the limitations of radiology in distinguishing mycetoma from other cystic pathologies of the trunk.

Definitive diagnosis hinges on the direct visualization of grains, and microbiological and cytological confirmation. Black grains are strongly suggestive of Madurella species in eumycetoma [1]. Treatment of eumycetoma is prolonged, often requiring several months of antifungal therapy [6]. Itraconazole remains the drug of choice because of its efficacy and tolerability [6]. Early diagnosis and initiation of treatment are important in preventing extensive tissue destruction and minimizing the need for surgical intervention [6]. However, in this case, long-term clinical response could not be assessed as the patient was lost to follow-up. Invasive procedures are generally reserved for cases with deep-seated or refractory lesions [6].

## Conclusions

This case highlights an unusual extra-pedal presentation of eumycetoma involving the posterior chest wall, initially mimicking a vascular malformation on imaging. It underscores the importance of maintaining a high index of suspicion for mycetoma in chronic subcutaneous lesions in endemic regions, even when arising at atypical anatomical sites. Early recognition and microbiological confirmation are essential to ensure appropriate antifungal therapy and to avoid unnecessary invasive interventions.
